# Non-Uptake of HIV Testing in Children at Risk in Two Urban and Rural Settings in Zambia: A Mixed-Methods Study

**DOI:** 10.1371/journal.pone.0155510

**Published:** 2016-06-09

**Authors:** Sonja Merten, Harriet Ntalasha, Maurice Musheke

**Affiliations:** 1 Department of Epidemiology and Public Health, Swiss Tropical and Public Health Institute, Socinstr. 57, 4002, Basel, Switzerland; 2 University of Basel, Petersplatz 1, 4003, Basel, Switzerland; 3 Department of Population Studies, University of Zambia, Great East Road Campus, Lusaka, Zambia; 4 Population Council Zambia Office, 4 Mwaleshi Road, Olympia Park, Lusaka, Zambia; London School of Hygiene and Tropical Medicine, UNITED KINGDOM

## Abstract

This article investigates reasons why children who were considered at risk of HIV were not taken for HIV testing by their caregivers. Qualitative and quantitative data collected in Zambia from 2010–11 revealed that twelve percent of caregivers who stated that they had been suspecting an HIV infection in a child in their custody had not had the child tested. Fears of negative reactions from the family were the most often stated reason for not testing a child. Experience of pre-existing conflicts between the couple or within the family (aOR 1.35, 95% CI 1.00–1.82) and observed stigmatisation of seropositive children in one’s own neighbourhood (aOR 1.69, 95% CI1.20–2.39) showed significant associations for not testing a child perceived at risk of HIV. Although services for HIV testing and treatment of children have been made available through national policies and programmes, some women and children were denied access leading to delayed diagnosis and treatment–not on the side of the health system, but on the household level. Social norms, such as assigning the male household head the power to decide over the use of healthcare services by his wife and children, jeopardize women’s bargaining power to claim their rights to healthcare, especially in a conflict-affected relationship. Social norms and customary and statutory regulations that disadvantage women and their children must be addressed at every level–including the community and household–in order to effectively decrease barriers to HIV related care.

## Introduction

Global estimates indicate that 3.2 million children 0–14 years of age were living with HIV at the end of 2013, most of them infected through mother-to-child transmission during pregnancy, labour and delivery [1]. Despite evidence that early antiretroviral therapy can dramatically improve child survival [2–7], less than one quarter of all HIV infected children receive antiretroviral therapy [1]. Many HIV infections in children in Sub-Saharan Africa continue to be detected and treated at a late stage of disease progression, despite the roll-out of PMTCT programmes and early HIV testing of infants [4, 8]. Health system constraints [9–11] and socio-cultural barriers [12–16] continue to curb the timely uptake of HIV testing of children.

In Zambia, a southern African country where over a million of people are living with HIV, about 100’000 children were estimated to be living with HIV in 2014 [17]. According to the Zambia Consolidated Guidelines for Treatment and Prevention, all children living with HIV below 15 years of age are eligible for free antiretroviral therapy. However only 45520 were actually receiving it, with numbers declining since 2013[18]. The rates of infants born to HIV infected mothers who were tested within two months of delivery (37.1%)[18] were also lower than expected. Early detection of an HIV infection in infants is important as without treatment; about half of the infected children die within the first two years of life, and 75–80% before the age of five [19]. In Zambia despite available testing opportunities, many of the infected children are identified after showing clinical symptoms which is considered late. [20, 21].

Health system constraints play an important role for delayed or not utilizing antiretroviral therapy. While rates of testing have been shown to be high at six weeks after delivery, only a minority of women come back for a second test or to obtain results. The turnaround time of HIV testing can be longer than 3 months, and many mothers do not return to the facility to obtain the results [9]. Failure to test might also be the consequence of: a lack of clear information provided to mothers, a mother’s inability to meet transport costs, opportunity costs, or of caregivers’ fears of negative social repercussions of caring for a child living with HIV [12, 15, 21–25].

Despite the availability of effective treatment, fears of the social and personal consequences of being HIV positive have contributed to reduced HIV testing among adults as well [26–37]. The stigma associated with utilizing testing might however be lower for children. A study from Zimbabwe suggests that testing of children is generally widely accepted in the population [38]. The availability of free antiretroviral therapy in Zambia might also positively influence the acceptability of HIV testing. This paper addresses the question as to despite the increasing access to HIV-related services for children, the reasons why some caregivers still do not initiate HIV testing even though they perceive a child to be at risk of HIV. Using a mixed-methods approach the determinants of not utilizing HIV testing for children perceived at risk of HIV are investigated, and gender and socio-cultural barriers of HIV testing of children are explored more in-depth in four areas of Zambia, two urban (Lusaka and Mazabuka) and two rural (Southern Province).

## Methods

### Study design and study population

The findings are reported as part of a larger study addressing socio-economic and socio-cultural barriers to HIV-related care. A qualitative exploratory study was combined with a cross-sectional study in two rural (Mbeza and Chivuna) and two urban (Lusaka and Mazabuka) sites in central and southern Zambia. Between October 2010 and March 2011, a questionnaire addressing socio-cultural barriers of HIV testing was administered to a random sample of adults. The questionnaire addressed socio-economic and socio-cultural barriers to HIV-related care for adults and children [26, 39, 40]. This paper analyses data from a subsample of caregivers living with an HIV risk child who were recruited at HIV clinics and health facilities/TB corners offering antiretroviral therapy were included in this nested substudy. The caregivers needed to be above 18 years old andliving with children <15 years of age in their home (own and fostered). Only caregivers who knew or considered that one of these children was HIV positive were included in this substudy.

### Qualitative data collection

Caregivers of children living with HIV were interviewed in a qualitative research component that was conducted prior to the cross-sectional study. We conducted three focus group discussions with ten caregivers of HIV positive children each (30 in total) in the rural and urban areas, and twelve interviews with caregivers living with HIV. In addition, we interviewed several key informants about HIV testing and antiretroviral therapy services, including health professionals and ART counsellors drawn from public sector health facilities. Participants were recruited mainly through community health workers or in health facilities in urban and rural sites. Focus group discussions were conducted by the researchers and a moderator. In-depth interviews were conducted in the homes of the participants or in a quiet place, for example near the health facility where confidentiality could be guaranteed. Most interviews were conducted in the local language (ciila or citonga) by either a researcher or with the assistance of a translator. All research assistants who were involved in conducting qualitative interviews had previously been formally trained as lay HIV counsellors. Most qualitative interviews were conducted between October 2009 and March 2010.

### Survey instrument and data collection

In the survey, specific questions were used to identify caregivers of HIV positive children, and of children who were suspected of being HIV positive. All persons living with children <15 years of age in the household were asked: if they knew or had ever suspected that a child in their household was HIV positive; their relationship with this child; whether the child had been tested for HIV; and if applicable, reasons for testing or not testing the child. The questionnaire further addressed socio-demographic factors (age, education, sex, religious affiliation, household size and composition, place); socio-economic variables (main source of income, relative wealth, food security); intra-household decision-making and responsibilities (decision-making on household expenditure and treatment-seeking, financial responsibilities); individual participation in social activities (religious and others); indicators of social cohesion in regards to the family (family and couple relations in terms of familial conflict, confidence and support); and tolerance of interpersonal violence (against women, if a family member misuses money); and social cohesion in the community (neighbourhood relations, social control, anticipated support, anticipated and enacted HIV stigma) [26]. Social cohesion is understood here as: “the absence of social conflict and the presence of strong social bonds, including institutions of reciprocity, of conflict management, and high levels of trust, measured at the community level” [41]. Interviews were conducted in the local language by trained interviewers between September 2010 and February 2011.

### Survey data analysis

The survey analysis included 304 caregivers from 59 communities who either took care of an HIV positive child, or who perceived a child in their custody at risk of being HIV positive. Factors associated with not utilizing HIV testing as the dependent variable were explored. Independent variables encompassed: child health and custody, individual socio-demographic variables at the level of the caregiver, as well as income generation and expenditure, individual social capital, decision-making power, health status and HIV-related beliefs. At the household level, food security, family cohesion including conflicts about money, material support, and tolerance of violence were included. At the community level we included neighbourhood support, social control, and levels of stigma and discrimination. First, mixed-effect univariable logistic regression analyses were conducted including a random effect for the sampling location. Random effects were only included if they significantly contributed to the model. Each variable was then tested for interactions with gender. Main effects with P-values <0.2 were entered in a comprehensive multivariable model for both outcomes. All variables with P-values <0.2 were retained for inclusion in multivariable models. In a second step, multivariable models were built by successively eliminating variables with p > 0.1. Due to the limited number of observations in each group, potential final models acknowledging model restrictions were compared using the Akaike Information Criterion (AIC) and the model with the lowest AIC was retained. Stata V.13.0 was used for statistical analysis.

### Qualitative data analysis

Interviews were audio-recorded, translated and transcribed verbatim. The narratives were then imported into Atlas-ti V.6, coded and interpreted using a latent content analysis approach [42]. For this paper all interviews with caregivers were coded and analysed again. First, open coding was applied. Codes were then grouped according to manifest and latent themes for further analysis. The findings were triangulated with the results from the survey.

### Ethical considerations

All participants were asked for written informed consent and thumb stamps were obtained for those who could not read and write. Ethical approval was obtained from the Ethikkommission beider Basel, Switzerland, and the University of Zambia Humanities and Social Sciences Ethics Committee. Administrative approval was also obtained from the Zambian Ministry of Health. Qualitative interviews were conducted by research assistants who had formerly been trained as lay HIV counsellors and were acquainted with the local health service offers. The survey was designed by teams of research assistants including health professionals and lay counsellors who could provide relevant information if requested. Information sheets with addresses of health facilities providing HIV testing and counselling for adults and children were routinely given to all participants. If requested, counselling by a trained professional was offered.

## Survey Results

The majority of the 304 caregivers of a child with confirmed or suspected HIV infection were HIV positive themselves (76.3%), female (80.6%), married (58.6%) and below 40 years of age (63.7%). One third of the respondents (33.5%) had more than 7 years of schooling. A small majority of respondents lived in rural areas (53.0%), and less than one in five respondents were in formal employment (18.1%). Two thirds of the respondents reported of having an own child being HIV positive or at risk (67.1%). The other cases were usually foster children living with a relative, either orphans (23.7%) or with parents still alive (9.8%). The average age of the affected children was 6.7 years (Table 1).

**Table 1:** Characteristics of caregivers and children perceived at risk of HIV; N = 304.

Characteristics	N	%
HIV positive respondents	232	(76.3%)
Female respondents	245	(80.6%)
Mean age	38 years	
Less than 3 years of school	23	(7.6%)
More than 7 years of school	102	(33.5%)
Married	178	(58.6%)
Urban respondents	143	(47.0%)
Employed, skilled and unskilled	55	(18.1%)
Own child	204	(67.1%)
Orphan	72	(23.7%)
Mean age of children considered at risk	6.7 years	
Child tested	266	(87.5%)
Child HIV positive	231	(76.0%)

One in eight children in the study population had not been tested for HIV despite a suspected HIV infection. Surprisingly the testing rate was not higher among mothers of young children who were themselves detected HIV positive during their last pregnancy. The majority of the children who underwent a test were actually seropositive (231 of 264). Testing was initiated primarily because of ill-health of the child or with parents/caregivers suspecting an HIV infection as the underlying cause (58.7%). Another quarter was detected positive during provider-initiated testing in a routine setting, with parents/caregivers not suspecting an HIV infection at first (25.6%). Most of the remaining children were taken for testing by their caregivers because one or both parents had died from AIDS (13.7%).

Caregivers who did not have the child tested were asked as to the main reasons for not utilizing testing. Fears of the reactions of the family (28%), or to be considered HIV positive oneself (22%), or a disagreeing spouse (20%) were the three main reasons given (Table 2). Only 12.0% of respondents who suspected a child of being HIV positive said that they did not know where to take the child for testing.

**Table 2:** Reasons for not utilizing HIV testing for a child perceived at risk.

Main reason for not utilizing HIV testing	N	%
Fear of family reaction	14	28.0
Fear that others think I am HIV positive	11	22.0
My spouse doesn’t agree	10	20.0
I am not responsible for this child/unknown	7	14.0
No testing options nearby/known	6	12.0
Bewitchment is cause of the child’s illness	2	4.0

We were interested in whether the stated reasons for not utilizing the HIV testing would be mirrored in low levels of family and couple cohesion among the non-utilizers and experiences concerning stigma. For this purpose, associations of family and couple cohesion and other relevant factors with non-utilization of HIV testing of children perceived at risk were calculated and are presented in Tables 3 and 4.

**Table 3:** Factors associated with non-uptake of HIV testing in children perceived at risk of HIV. Univariable mixed-effect logistic regression^a^.

	Odds Ratio	95% CI		P-Value ^b^
**Child-related factors**				
Child often sick	0.23	0.11	0.51	**0.000**
Orphan	1.73	0.79	3.40	0.171
**Caregiver-related individual factors**				
Caregiver female	0.71	0.27	1.82	0.472
Caregiver age	0.99	0.96	1.03	0.608
More than seven years of school	0.45	0.18	1.14	0.091
Widowed or divorced	0.34	0.07	1.64	0.182
No ownership of assets	5.00	1.26	19.83	**0.022**
Cannot decide over income	1.34	0.78	2.32	0.291
Impaired health ^c^	1.52	0.98	2.36	0.060
Fears HIV-related stigma ^c^	1.39	1.10	1.75	**0.006**
**Household-level factors**				
Food insecurity ^c^	1.46	0.94	2.27	0.091
Household poorer than neighbours ^c^	1.37	0.92	2.05	0.056
Tolerance of violence score ^c^	1.61	1.10	2.37	**0.015**
Conflict in couple/family ^c^	1.61	1.17	2.22	**0.004**
**Neighbourhood-level factors**				
Low social cohesion, household and neighbours ^c^	1.53	1.02	2.30	**0.039**
Stigmatized HIV+ children in neighbourhood ^c^	1.60	1.11	2.32	**0.012**

^a^ A random effect was integrated for the place of residence of the respondent.

^b^ P-values <0.05 are represented in bold

^c^ measures unit increase of score 1–5

**Table 4:** Factors associated with non-uptake of HIV testing in children who are considered at risk for HIV. Multivariable logistic regression analysis. Model with lowest AIC.

Model 1^a^	Odds Ratio	95% CI ^b^		P-Value
Child often sick	0.25	0.12	0.53	**0.000**
Household poorer than neighbours ^c^	1.53	0.97	2.41	0.070
Conflict in couple/family of caregiver ^c^	1.35	1.00	1.82	**0.048**
Stigmatized HIV+ children in neighbourhood ^c^	1.69	1.20	2.39	**0.003**

^a^ Pseudo R2 = 0.1459

^b^ Robust standard errors adjusted for clustering on location

^c^ OR for increment 1 in a scale of 1–5

In the univariable analysis (Table 3) poor child health (OR 0.23, 95% CI 0.11–0.51) was a protective factor; those with sickly children were more likely to report testing. Non-uptake of testing increased sharply if a caregiver did not own any household assets (OR 5.00, 95% CI 1.26–19.83). Caregivers’ HIV-related beliefs influenced uptake of testing as well. Fears of HIV-related discrimination were associated with not-testing a child for HIV (OR 1.35, 95% CI 1.04.1.74).

Social and relational factors were associated with uptake of testing in several ways. Tolerance of violence decreased the likelihood that a child was tested (OR 1.61, 95% CI 1.10–2.37). Conflicts between the couple or within the family (OR 1.61, 95% CI 1.17–2.22), experienced discrimination of HIV positive children in the neighbourhood (1.60, 95% CI 1.11–2.32), and low support and cohesion in the neighbourhood (OR 1.53,95% CI 1.02, 2.30) were associated with not -testing a child for HIV as well.

The multivariable models showed that gender, education, fears of HIV-related stigma, no ownership of assets and tolerance of violence in the household were no longer significantly affecting the outcome once controlled for household-level factors. A child’s health status remained the most important factor for uptake of testing. Not utilizing HIV testing was much less common among children who were often sick (aOR 0.25, 95% CI 0.11–0.61). In contrast, having conflicts between the couple or within the family (aOR 1.36, 95% CI 1.00–1.82), and being poorer as compared to the neighbours (aOR 1.53, 95% CI 0.97–2.41), limited the testing of a child, although the latter was only marginally significant. Observed stigmatisation of seropositive children in one’s own neighbourhood was clearly a problem for the utilization of HIV testing (aOR 1.69, 95% CI1.20–2.39).

## Qualitative Study Results

The complementary qualitative results shed light on how conflicts in the family unfold due to an HIV diagnosis of a child and point at the gendered expressions of stigma in times of ARV availability.

### Reputation and men’s decision-making power

Reactions of denial towards a possible infection were common when the dynamics between a couple were such that men dominated decision-making and at the same time feared to lose their reputation through being associated with HIV. Men’s denial strategies, and the belief that they need to be in control over their wives’ and children’s actions had implications for their children’s health, as several respondents pointed out:

“We asked the husband of [an HIV positive pregnant woman] to come with her [to the clinic], but he never did. When she delivered, we gave her Septrin for the baby. But the husband refused that the baby take the medicine, and said ‘Not my child, I do not want.’ Men only come forward for the test when they are very sick themselves.”

(Health professional, Chivuna)

Another woman we interviewed, who was diagnosed with HIV during her last pregnancy, told us how she negotiated the child’s therapy with her husband who initially refused that his child would be treated with antiretrovirals:

“My husband was getting annoyed [when he was informed that the child was supposed to start ART]. I said to him ‘since the child is yours I won’t commence it on ARVs. But I myself will take the ARVs.’ Then after I started taking the ARVs he said ‘get also the child and commence it on the ARVs. …. [over a year later] my husband was sick … and finally he asked me to escort him for VCT. Thereafter he was thanking me, also for having saved my son and myself”.

(Woman, Mbeza, Kafue Flats)

Controlling behaviour, rejection and emotional violence were reported to be common practice among men who tried to blame their wives for bringing HIV into the home:

“Sometimes [men] would go for a test themselves elsewhere and start taking the drugs clandestinely, without telling the wife. When the wife does the test and tells him of the test, he would accuse her openly of being unfaithful and would want her go packing out of the marital home. Women are really scared of these men. …

You find men threatening their wives and saying ‘If you go to the clinic do not come back, that is the end of our marriage’.”

(Health professional, Chivuna)

### Economic dependency

Some women strongly depended on their husbands’ financial resources, which occurred not only among the very poor. A woman from Lusaka who lived in a lower middle-class neighbourhood, married to the owner of a medium-size enterprise told us how she had been reluctant to test for HIV partly because it was not well accepted in her church and because her husband did not want to be tested either. During her first six pregnancies she never went for HIV testing during ante-natal care visits. Only after the delivery of twins when she and one child were severely sick, she and her baby were tested in the clinic. Until we conducted the last interview with her, she said she hadn’t collected the results–or maybe she hadn’t disclosed them to us and her family.

“I have been tested and they tell me ‘you should come back with your husband’, but when I told my husband he didn’t even answer me, he just kept quiet, so now I don’t know whether he is going to agree. Since I already went for VCT, now when the results come, what I’m going to tell him, and how am I going to tell him?”

(Woman, Lusaka)

She had told us also about her economic problems in that she was fully dependent upon the husband with her children. There were many stories about divorce and separation once one of the partners was tested HIV positive; children were separated and split between the families which instilled fear in many married women. When a couple was divorced, a woman usually had to seek support from her own relatives. In some cases, however, families were far away, fragmented, and poor themselves. In the rural area of the Kafue Flats we met a woman in a polygynous household whose husband did not provide her with food and the necessary means to support her children although he owned cattle and could have afforded it. He only gave her usufruct right to land where she planted maize for consumption in the home. Her children had been sickly for years, and one had died allegedly from AIDS at a time antiretroviral therapy for children had already become available for free. However, she usually took her children to the herbalist–with cost implications–instead of the nearby clinic. Later on she told us that she did not want to be tested for HIV when the children were sick because she feared to be blamed as the one who brought HIV into the marriage. As she lived in a precarious situation, being food insecure and lacking a family network to support her and her children, risking the thin support she received by her husband and co-wives would have brought her in an even more difficult situation. Only when she, her co-wife and her husband were all severely sick, she went to the clinic where she was finally tested for HIV and started antiretroviral therapy. Now she couldn’t be blamed as the one who brought the infection into the marriage. Thereafter, she also took her surviving children to the clinic where they were all tested and treatment was initiated.

### Stigma

Besides economic constraints, fear of open discrimination was still common, although there seemed to be differences between the rural and urban areas. Women were sometimes affected by stigma through the health status of their children:

“When I stopped breastfeeding my child, who was sick, a lot of people, including my friends, where talking about the reason why that child was sick. Maybe the mother is HIV infected. They didn’t try to help me. They were not even holding the baby; they didn’t want to touch it. Only when they saw that the baby’s health improved, they thought that maybe it was just an ordinary illness. There is still a lot of stigmatisation in the compounds. They said they thought it was AIDS. I was saved by the child who looked healthy. Otherwise I would have been heavily stigmatised in the compound.”

(Woman, Ndeke compound, Mazabuka)

Stigmatization of older children was feared as well so that mothers would prefer to hide their HIV status:

“I don’t want my child to be embarrassed. It is better people know only when he grows up. Only for now, I don’t want anybody to know. I can just choose some persons who can be trusted and tell them.”

(Woman, Shikapande village, Mbeza)

Older children were facing stigmatizing attitudes in the communities, too.

“Parents, when advising their children, would say: Don't play with that friend of yours because he or she has a virus’. … So you find children when they are playing; they would [do as informed by] their parents who said ‘don't play with your friends because they are HIV positive’.”

(Man, Ndeke compound, Mazabuka)

In one of the focus groups of men living with HIV (Mazabuka), the participants confirmed that stigmatizing attitudes were a barrier for some parents to take their children for testing. They spoke of a case in their community where the parents wouldn’t take their sick child to the clinic, allegedly because they didn’t want it to be tested for HIV, emphasizing the key role husbands could play in such cases.

### Responsibility

Children are however not only seen as passive dependants. Already at an early age they are considered old enough to look after their own health. It was not rare that at ten years or sometimes even at a younger age, children who were moderately sick or had to go for a follow-up visit to the clinic were not accompanied by an adult. For HIV testing, too, children were considered old enough to go on their own behalf at an early age:

“A child [who is] able to reason; it’s the duty of the child to take himself [for an HIV test]. Children who are only nine years and below we take them; we force them to go.”

(Woman, Ndeke compound, Mazabuka).

Children and adolescents were perceived as having the capacity to take such a decision on their own. At the same time this meant that they may refuse HIV testing, as an elderly woman who took care of three of her grandchildren told us:

“I decided that [my granddaughter] should have an HIV test because she was very sick. But she refused and I could not force her to have an HIV test… I suspect that perhaps because they know their movements [meaning involvement with men] so they are scared that perhaps the test will be reactive”.

(Woman, Ndeke compound, Mazabuka)

## Discussion

Delays in treatment-seeking for children who were considered at risk to be HIV positive occurred out of economic, social and personal reasons on the side of their caregivers. These have been outlined in the previous sections. We will now synthesize those by first referring to common narratives of barriers to care, which fall into the socio-ecological model of individual, family, society, health system and structural determinants [43]. By drawing upon a human rights based approach, we will then try to integrate these narratives into a broader discussion of the observed tension between universal access to healthcare on the one hand and the lack of recognition of women’s and children’s rights to use these healthcare services on the household level on the other hand.

### Personal and couple-related factors

Anticipated couple conflicts, fear of rejection and partner stigma emerged as major themes for non-disclosure and non-use of HIV testing and treatment for children, which has been observed in many other studies [12–16, 24, 44–49]. It is important to distinguish HIV-related stigma from pre-existing couple conflicts. An HIV diagnosis–like any other STI diagnosis–will likely raise the tangible question of who brought the infection to the couple. The connotation of immorality and death assigned to HIV, which is at the core of the stigma of HIV [50, 51], can reinforce pre-existing conflicts or distrust. In such situations, devaluating attitudes and other forms of emotional or physical violence motivated by HIV may be a way to shift blame to the partner in relationships that are already characterized by conflicts and unequal power-relations, and may ultimately lead to the breaking up of a relationship. In our study, women who had conflict-affected partnerships were in fact more hesitant to take a child for testing. Ethnographic research has showed that the strength of the affective relationship between family members influences whether a person living with HIV is cared for or not up [46]. In these cases, couple testing and counselling services are likely to be a good way to support couples. In cases however, where conflicts pre-exist, and especially if there is economic dependency within the relationship, other support strategies are required that mitigate the economic impact of an eventual separation and divorce for the economically weaker caregivers and their children.

### Household economy

Economic factors were barriers to accessing care in our study despite the free HIV-related care in the health facilities. While distance to health facilities and related transport costs were found to be a direct barrier to care in previous studies in rural areas of southern Zambia [21], in our study these were rarely stated as reasons for not utilizing HIV testing services. This might be due to the increasing availability of nearby testing sites also in more remote areas in the recent years. However, living in a poor household was found to be a barrier to the testing of children, although the association was only marginally significant in the quantitative study. Economic dependency of women, which acted more indirectly in the form of fear to lose material support in case of conflict due to a disclosed HIV infection, was an important narrative in the qualitative interviews in rural and urban settings. Economic dependency affected the rural and urban poor but equally middle-class urban women were not necessarily economically autonomous. Household prosperity was not a guarantee for adequate access to services should the woman fear rejection and separation. This may partly explain why in the quantitative analysis poverty was only marginally associated with the HIV testing of a child.

### Stigma and reputation in the community

Stigma and fear of social rejection have been described as main barriers for HIV testing of children throughout the region [13, 15, 24, 43, 48, 52–54]. Observed stigmatization of children in the community was a barrier to the testing of children in our study as well. It is plausible that caregivers would want to protect their children from discrimination and marginalisation, as it was also stated in one of the interviews. Stigma was anticipated and internalized, jeopardizing treatment-seeking and disclosure to persons who might otherwise provide support.

Among men, the reluctance to accept couple HIV testing or the testing and treatment of a child seemed also related to fears of losing reputation in the public sphere. Among women the fear of divorce was the overriding theme which, however, also went along with a loss of reputation in the community.

### Responsiveness of the health system

As many HIV infections in children still go unnoticed, various strategies have been developed on the side of the health system to detect infections early. Several studies in the region have shown that provider-initiated testing can be highly successful in detecting HIV infections in hospitalized and outpatient children [10, 55–58] Early infant diagnosis during postnatal care has increased with community-based interventions, for example [59], or through the use of cash transfers to women who accept available PMTCT services and remain in care [60]. Our results confirmed that provider-initiated HIV testing and counselling in clinical care once children are sick were an opportunity to detect HIV infections that previously went unnoticed, and where caregivers were not aware or willing to initiate HIV testing. In Zambia, most caregivers knew where to take a child for HIV testing where HIV testing, counselling and antiretroviral therapy were widely available for free [61], and mobile and home-based voluntary counselling and testing programs were ongoing in some of the study areas. An earlier qualitative study however had found deeply rooted distrust in ARV programs in the same study area [62] and during the time of the data collection, newspapers spread rumours about medical trials deliberately infecting women with HIV. While distrust in the health system did not seem to affect treatment seeking of children in our study population, qualitative research in urban areas had shown that many people, nonetheless, preferred to opt for alternative therapy or faith healing in which they confided more [63].

### A rights-based perspective

Human rights- based approaches for health acknowledge poverty and related ill-health as consequences of structural disempowerment and exclusion, and call for action to address the structural dimensions of human rights violations [64]. Human rights also provide the legal standards for universal health coverage, framing government responsibilities and respective health policies in order to providing equitable healthcare also to vulnerable populations [65], Free healthcare or social cash transfers are examples to address inequities in access to healthcare. In Zambia, user fees were only introduced in the early 1990s to be abolished again in 2006–7 for all primary healthcare services [66]. ince then, services for HIV testing and treatment were made available for free throughout the country. At the end of 2014, Zambia additionally launched a new National Social Protection Policy to alleviate poverty among the very poor. The policy foresees cash transfers to the most vulnerable households, including single women and orphaned children, to guarantee sufficient income security to meet basic needs. [67].

Our findings point to the limits of these approaches as structural inequalities continue to produce inequalities within society. Despite free HIV testing and treatment some women and children are denied access to HIV-related services–not on the side of the health system, but on the household level, as a consequence of women’s and children’s subordinate positions. Social norms, assigning the male household the power to decide over the use of healthcare services by his wife and children, are still sustained by unequal laws and practices, such as the unequal customary marriage rules, which puts women and children at a disadvantage with regards to property rights. For example, in case of a divorce, women are usually not entitled to anything from the man’s property [68]. Women’s bargaining power to seek healthcare against the will of their husbands is therefore often low especially for women who are not economically autonomous. This is only partly mitigated by service-delivery interventions, such as home-based counselling or testing, or by economic empowerment through micro-credit or social cash transfer programmes, which in general have a beneficial effect on various child health outcomes [69]. Yet in case of couple conflicts, some women may not be able to make use of financial incentives and social protection programmes as long as men are not committed to provide support.

Service providers and policy-makers deal with couple conflicts primarily as a private issue located outside the realm of public health. HIV-related policies and programs are not prepared to deal with gendered hierarchies in households despite the many health consequences this can have. Gender inequality shapes these conflicts often to the disadvantage of women who are expected to not question their male partner’s decisions. Research on child health has long identified gender inequality as a barrier to healthcare [42, 69, 70], and macro-level analyses confirm the detrimental effect gender inequality can have for child health, leading to increased neonatal, infant and child mortality [71]. Male involvement in reproductive and child health has been promoted as one possible way to achieve men’s commitment to child healthcare, but it bears the risk of shifting decision-making back to men.

If a caregiver–male or female–precludes a child from using health services we may want to look at it from a rights-based approach as well [65, 71]. If we acknowledge women’s and children’s right to health as a human right, we must consider it as indisposable; it exists in its own right [64] and as such is independent from the consent of a family head. Any discriminatory decision-making based on gender or age-related social inequality, which jeopardizes equitable access to healthcare, should equally be addressed by public health interventions if the right to health for women and children is to be turned into actual use of appropriate services. Legal frameworks and practical jurisdiction need to challenge all dimensions of gender-based inequality, including in marital unions. While financial constraints can be a barrier to accessing healthcare in many cases, HIV testing and treatment were available for free in the study areas. Men, as well as women, who restrict access to HIV-related care of their family members, should be sensitized towards the basic right of every person to use these services, and mechanisms to improve justice should be considered.

This study has several possible limitations. The survey results cannot be generalized to the population of caregivers who were recruited at health facilities, and the type of health service delivery varied between areas. The qualitative data were specific to the study areas, however, the findings allow nonetheless for a broader discussion about gaps in policies and programs. There is a need for further applied research on how best to support individuals in their right to access health services when they face the possible breakdown of their family, in psychosocial and economic terms.

## Conclusions

Economic constraints, dependency, fear of stigma, fear of losing reputation, and gender hierarchies in the household contributed to delays in treatment-seeking for children who were perceived to be at risk for an HIV infection. While free healthcare facilitates access to healthcare for economically weaker groups in the population, community-centred interventions remain important to address stigma and gender and rights themes. As long as women are in a subordinate dependent position once they are married, their bargaining power will be limited to claim their rights to healthcare, especially in a conflict-affected relationship. This inevitably affects child health as well. Social norms and customary and statutory regulations that disadvantage women and their children must be addressed at every level–including the community and household–in order to effectively decrease barriers to HIV related care.

## Supporting Information

S1 Dataset (DTA)
